# Circadian Disruption and Consequences on Innate Immunity and Inflammatory Response

**DOI:** 10.3390/ijms232213722

**Published:** 2022-11-08

**Authors:** Viera Jerigova, Michal Zeman, Monika Okuliarova

**Affiliations:** Department of Animal Physiology and Ethology, Faculty of Natural Sciences, Comenius University, 842 15 Bratislava, Slovakia

**Keywords:** circadian rhythms, chronodisruption, inflammation, innate immunity, light at night, phase shifts

## Abstract

Circadian rhythms control almost all aspects of physiology and behavior, allowing temporal synchrony of these processes between each other, as well as with the external environment. In the immune system, daily rhythms of leukocyte functions can determine the strength of the immune response, thereby regulating the efficiency of defense mechanisms to cope with infections or tissue injury. The natural light/dark cycle is the prominent synchronizing agent perceived by the circadian clock, but this role of light is highly compromised by irregular working schedules and unintentional exposure to artificial light at night (ALAN). The primary concern is disrupted circadian control of important physiological processes, underlying potential links to adverse health effects. Here, we first discuss the immune consequences of genetic circadian disruption induced by mutation or deletion of specific clock genes. Next, we evaluate experimental research into the effects of disruptive light/dark regimes, particularly light-phase shifts, dim ALAN, and constant light on the innate immune mechanisms under steady state and acute inflammation, and in the pathogenesis of common lifestyle diseases. We suggest that a better understanding of the mechanisms by which circadian disruption influences immune status can be of importance in the search for strategies to minimize the negative consequences of chronodisruption on health.

## 1. Introduction

Circadian rhythms (circa = about; dies = day) represent endogenous oscillations with a period of approximately 24 h. In most species, circadian rhythms are effectively entrained by external factors, primarily by a light/dark (LD) cycle, allowing the anticipation of daily periodic changes in the environment [1,2]. Mammalian circadian rhythms are governed by a master clock located in the suprachiasmatic nuclei (SCN) of the hypothalamus. The SCN receives photic input from the environment and transmits the information to peripheral oscillators to coordinate the optimal timing of physiological and behavioral processes [3].

Life on Earth has evolved under relatively stable conditions of bright days and dark nights. The sun is the primary light source for the majority of organisms, with daylight illumination varying from 50,000 to 100,000 lx, and low illuminance levels during the night, reaching up to 0.3 lx at the full moon [4,5]. Nowadays, light exposure is no longer limited by the natural LD cycle in the industrialized world. Recent studies show that more than 80% of the world’s population lives in light-polluted areas [6] and increasing exposure to artificial light at night (ALAN) represents a novel challenge for both humans and wildlife [7,8]. The straightforward impact of compromised LD cycles is linked with circadian disruption, which can be manifested at multiple levels, depending on the nature of mistimed light information. Such situations are a common part of modern society and include especially various shift work schedules, time-zone transitions, or unintentional ALAN exposure. Here, circadian disruption refers to transient or chronic misalignment between the external LD cycle and endogenous circadian clocks, which can further lead to internal misalignment (impaired phase relationships) or desynchronization (changes in period) among individual endogenous rhythms, diminished peak-trough differences in these rhythms (changes in amplitude) or complete arrhythmicity [9]. The main result is attenuated or abolished circadian control of important physiological processes, underlying potential links to adverse health effects [10,11]. Many epidemiological studies examining the risk of common lifestyle diseases among shift workers or due to ALAN found a positive correlation with the incidence of sleep disorders [12,13], cancer [14,15], metabolic and cardiovascular diseases [16,17,18]. A common feature of most lifestyle and chronic diseases is low-grade inflammation, which can further potentiate disease progression [19]. Therefore, a better understanding of the mechanisms by which circadian disruption influences the status of the immune system and inflammatory responses can be of importance in the search for strategies to minimize the negative consequences of environmentally induced circadian disruption on health.

In the current review, we document the effects of circadian disruption resulting from compromised LD information on fundamental aspects of the innate immune defense under homeostatic conditions, as well as in response to acute inflammation and in the pathogenesis of diseases. We focus on data obtained from experimental studies in rodents and first compare the immune consequences in transgenic animal models with genetic mutation or deletion of specific clock genes. In the following sections, we evaluate the impact of different disruptive LD regimes, particularly light-phase shifts, dim ALAN, and constant light (LL) on innate immune cells and their effector functions.

The literature search was performed in the PubMed and Google Scholar databases based on the following keywords: artificial light at night, circadian disruption, constant light, dim light at night, innate immunity, inflammation, jet lag, macrophages, monocytes, neutrophils, NK cells, shift work. Relevant papers were evaluated by title and abstract, followed by a full-text overview.

## 2. Mammalian Circadian System

In mammals, circadian timekeeping is organized into a multi-oscillator system operating in a hierarchical manner, with the SCN as a master oscillator [20]. The SCN neurons are located alongside the third ventricle above the optic chiasm and form a unified circadian network [21]. Light information is perceived by the intrinsically photosensitive retinal ganglion cells, containing the photopigment melanopsin, and conveyed via the retinohypothalamic tract into the SCN [22]. Subsequently, the SCN communicates timing information to individual peripheral oscillators via neural and humoral pathways [23].

At the molecular level, circadian rhythms are generated through transcriptional-translational feedback loops of clock genes and their protein products, forming a basis of the self-sustained and cell-autonomous molecular clocks [24]. The core feedback loop consists of positive and negative regulators. The CLOCK and BMAL1 proteins heterodimerize to form the CLOCK/BMAL1 complex, which activates transcription via binding to E-box enhancer elements in the promoters of clock genes, Period (*Per1*, *Per2*, and *Per3*) and Cryptochrome (*Cry1* and *Cry2*). The PER and CRY proteins represent a negative limb of the loop, as they form the repressive PER/CRY complex, which enters the nucleus, combines with CLOCK/BMAL1, and inhibits the transcription of E-box-controlled genes [25]. The availability and stability of PER and CRY proteins are regulated by protein kinases and phosphatases [26].

Additionally, the core loop is stabilized by accessory feedback loops, consisting of transcriptional activators and repressors, which regulate target genes either through ROR response elements (RORE) or D-boxes [27]. In this way, nuclear receptors REV-ERBs (α/β) repress and retinoic acid-related orphan receptors (RORα/β/γ) activate the transcription of *Bmal1*, which contains RORE in its promoter. On the other hand, the CLOCK/BMAL1 complex can activate the transcription of genes encoding REV-ERBs [28]. The next feedback loop is formed by nuclear factor interleukin-3 (NFIL3, also known as E4BP4) and D-box binding protein (DBP), which competitively repress or activate the transcription of D-box regulated genes, such as those encoding the circadian proteins PER, REV-ERBs, and RORs [24]. Importantly, circadian regulatory elements have also been identified in the promoters of numerous immune genes, underlying direct crosstalk between the components of the molecular clockwork and the immune system [29,30].

## 3. Circadian Rhythms in Innate Immunity

Innate immune mechanisms represent the first line of defense against invading pathogens. Circulating and tissue-specific innate immune cells recognize pathogens or cell injury via pattern recognition receptors [31]. Subsequently, initiated signaling pathways induce the release of specific immune mediators, such as cytokines, chemokines, and antimicrobial peptides, which are involved in numerous effector functions [32]. Effective host defense against infection is based on tightly regulated immune processes. Inflammation is an essential part of the innate immunity in response to infection or tissue injury. However, deregulated inflammatory responses or disbalance between favoring and limiting factors can lead to chronic inflammation and tissue damage [19].

Immune functions, including innate immune mechanisms, are under circadian control. Leukocyte trafficking, inflammatory responses and susceptibility to pathogens exhibit their peaks and troughs at specific times of the day [33,34]. In steady state, circulating immune cell numbers reach a peak during the day in mice and rats [35,36] and during the night in humans [37]. High and low leukocyte numbers in the blood over 24 h mirror their mobilization from the bone marrow in the passive phase (light phase for rats) and their recruitment to tissues at the onset of the active phase (dark phase for rats) [38]. Leukocyte oscillations persist in an absence of external entraining cues, such as the LD cycle, thereby indicating their endogenous nature [39,40]. Rhythmic leukocyte trafficking is complementary controlled by extrinsic factors, including neural and humoral outputs of the central oscillator, immune cell-autonomous clocks, and tissue-specific microenvironment [35,41,42]. For example, reported data show that β3-adrenergic signaling in the mouse bone marrow down-regulates C-X-C motif chemokine ligand 12 (*Cxcl12*) expression during the light phase, controlling the rhythmic release of hematopoietic progenitors from the bone marrow into the circulation [43]. Additionally, low corticosterone levels at the onset of the light phase allow proliferation of hematopoietic cells and contribute to their egress into the circulation [44].

The exit of leukocytes from the circulation to the tissues is facilitated by coordinated interactions between adhesion molecules on the endothelium and the surface of leukocytes [45]. In general, rhythmic expression of adhesion molecules, such as intercellular adhesion molecule 1 (ICAM1), vascular cell adhesion molecule 1 (VCAM1), and selectins on endothelial cells promotes time-of-day-dependent leukocyte transmigration into the lymphoid and non-lymphoid tissues [35].

Susceptibility of the immune system to bacterial, viral, and parasitic infections varies across 24 h [46]. One of the first evidence was provided by the experiment, in which mice were administrated a lethal dose of lipopolysaccharide (LPS). An immune challenge given at the end of the rest period led to a mortality rate of 80%, whereas the same LPS dose given in the middle of the active period resulted in a mortality rate of only about 20% [47]. A subsequent study demonstrated that this time-of-day-dependent mortality rate following LPS administration correlates with the increased cytokine response at the end of the light phase (ZT11; ZT—Zeitgeber time) compared to the dark period (ZT19) [48]. Daily variation in susceptibility to inflammatory challenge has also been shown to correlate with nuclear factor kappa B (NF-κB) activation, as mice administrated with a toll-like receptor (TLR) 5 ligand in the middle of their passive phase (ZT6) displayed higher NF-κB activation compared to mice injected in their active phase (ZT18) [49].

Macrophages represent one of the main sources of pro-inflammatory cytokines, and their inflammatory response is controlled by the circadian clock [50]. Mouse peritoneal macrophages show higher LPS-induced expression of inflammatory cytokines, mainly interleukins *Il-6*, *Il-12b*, and chemokines *Cxcl1* and C-C motif chemokine ligand 2 (*Ccl2*), when isolated at the end than at the beginning of the subjective passive phase [51]. Moreover, the rhythm of inflammatory monocytes Ly6C^high^ in the blood corresponds with the time-of-day-dependent immune response to *Listeria monocytogenes* infection, reflected by higher levels of CCL2 in the serum and peritoneal fluid upon the induction of infection at ZT8 compared to ZT0 [52].

Neutrophil infiltration into the skeletal muscle was increased upon tumor necrosis factor-alpha (TNFα) challenge at the beginning of the active phase (ZT13) compared to the passive phase (ZT5), and positively correlated with greater *Icam1* expression on the muscle endothelial cells [53]. On the other hand, in a mouse model of acute lung inflammation, the recruitment of neutrophils was promoted by the rhythmic release of chemokine CXCL5 from bronchiolar epithelial cells with higher levels upon LPS administration at the beginning of the resting phase compared to the active phase [41].

## 4. Effects of Circadian Disruption on Innate Immunity

Disruption of the circadian timing system can directly impact daily rhythms in the immune parameters, bearing potential negative consequences on the host’s ability to effectively cope with pathogens or tissue injury. Other complications can include a disturbed balance between anti- and pro-inflammatory mechanisms that can lead to either immunosuppression or promote a pro-inflammatory microenvironment favorable for chronic inflammatory diseases.

The following sections will evaluate the abovementioned ways, in which innate immune cells can respond to circadian disruption induced by the targeted deletion of individual clock genes or by exposure to disruptive LD regimes, including light-phase shifts, dim ALAN, and LL.

### 4.1. Genetic Circadian Disruption

Many important functional interactions between components of the molecular clock and the immune system have been revealed using animal models with the deletion of clock genes at the systemic or cell-specific levels [29,30]. These studies show that individual clock proteins can differ in their pro-inflammatory and anti-inflammatory properties. Typical immune phenotypes associated with deficiency of the main circadian genes, including *Bmal1*, *Clock*, *Per1/2*, *Cry1/2*, *Rev-erbα*, *Rorα*, and *Nfil3*, are presented in Table 1.

BMAL1 is a central component of the mammalian molecular clock and plays a central role in circadian–immune interactions. Systemic deletion of *Bmal1* eliminated circadian rhythmicity in the central pacemaker and periphery, resulting in a complete behavioral arrhythmicity [80,81]. *Bmal1^−/−^* mice also lost rhythmicity in the numbers of leukocytes and immature hematopoietic cells in the peripheral blood [43,54]. However, particularly models with targeted *Bmal1* deletion in myeloid cell lineages have revealed an essential role of BMAL1 in the control of the time-of-day-dependent effector functions of monocytes and macrophages. Mice with deletion of *Bmal1* in myeloid cells lost daily variability in circulating inflammatory Ly6C^high^ monocytes, showing higher susceptibility to *Listeria monocytogenes* infection [52]. In another study, myeloid *Bmal1*-deficient mice on the *Apoe^−/−^* background showed increased recruitment of Ly6C^high^ monocytes to atherosclerotic lesions with polarization to pro-inflammatory M1 macrophages [61]. In vitro experiments using bone marrow-derived macrophages (BMDMs) demonstrated that *Bmal1* deficiency amplified acute inflammatory response to LPS, as was manifested by enhanced production of pro-inflammatory cytokines, suppressed antioxidant pathways. and increased reactive oxygen species levels [57,58]. Surprisingly, myeloid *Bmal1* deficiency was also found to confer protection against pneumococcal infection that was attributed to increased motility and phagocytic activity of *Bmal1* deficient macrophages [60]. In neutrophils, specific deletion of *Bmal1* eliminated daily variability in granule content and neutrophil extracellular traps formation [62]. In general, the above-mentioned studies demonstrated the anti-inflammatory effects of BMAL1, which are probably mediated by CLOCK/BMAL1-dependent transcriptional regulation of genes containing E-box. For example, circadian monocyte trafficking is driven by time-of-day-dependent expression of chemokines (such as *Ccl2*), which are under the repressive transcriptional control of BMAL1 through recruitment of the polycomb repressive complex 2 [52].

In contrast to BMAL1, CLOCK protein has been shown to enhance NF-κB-mediated transcription and production of pro-inflammatory cytokines, and these effects were independent of the transactivation capacity of the CLOCK/BMAL1 complex on E-box containing promoters [49]. CLOCK was found in protein complexes with the p65 subunit of NF-κB and CLOCK overexpression was associated with enhanced NF-κB activation [49]. These findings were supported by reduced activation of NF-κB in response to immune challenge in mouse embryonic fibroblasts (MEFs), as well as hepatocytes of *Clock*-deficient mice compared to wild-type controls [49]. Similarly, reduced induction of pro-inflammatory cytokines upon LPS challenge has been observed in MEFs and BMDMs from *Clock*-mutant mice [63,64]. Moreover, day/night differences in inflammatory response to *Salmonella* infection were eliminated in the gut of *Clock* mutants [63].

Models with genetic disruption of clock genes *Per* and *Cry* have revealed distinct roles of these clock components in the regulation of immune functions. A study in *Per1* mutant mice showed that they maintained circadian expression of perforin, granzyme B, and interferon-gamma (IFNγ) in splenic NK cells, though these rhythms were either attenuated or phase-shifted [65]. On the other hand, in *Per2* mutant mice, serum IFNγ concentrations as well as mRNA and protein levels in the spleen completely lost daily rhythmicity [66]. These eliminated IFNγ rhythms can be translated to impaired IFNγ production by the splenic NK cells upon LPS challenge in *Per2* mutant mice, and suppressed response in serum IFNγ and IL-1β levels [67]. Moreover, this study found an increased survival rate of *Per2* mutants following a lethal dose of LPS compared to controls [67]. Mutation of *Per2*, disrupting the ability of PER2 to interact with other clock proteins, can also significantly affect TLR9-mediated immune responses, as peritoneal macrophages from *Per2* mutants showed reduced expression of *Tlr9* and decreased TLR9 ligand-induced production of TNFα and IL-12 [68]. In contrast to *Per2* defects, the absence of *Cry* genes leads to a pro-inflammatory phenotype. In *Cry1* and *Cry2* double knockout fibroblasts, enhanced constitutive expression of pro-inflammatory factors was observed, and this was mediated by the constitutive activation of NF-κB and protein kinase A (PKA) signaling [69]. The proposed mechanism shows that CRY1 can inhibit PKA-mediated phosphorylation of p65 through binding to adenylyl cyclase and suppression of cyclic adenosine monophosphate levels [69]. In this study, *Cry1^−/−^Cry2^−/−^* mice exhibited not only enhanced basal expression of *Il-6*, *Cxcl1*, and inducible nitric oxide synthase (*iNos*) in the BMDMs but also elevated cytokine responses to LPS compared to wild-type animals [69].

The nuclear receptors REV-ERBα and RORα represent important regulatory components linking the circadian and immune systems and exert mostly anti-inflammatory effects. Peritoneal macrophages isolated from global REV-ERBα knockout mice (*Rev-erbα^−/−^*) displayed augmented pro-inflammatory response to LPS [51,56,71]. Simultaneously, the absence of circadian rhythmicity in LPS-induced IL-6 response was demonstrated in the cultured *Rev-erbα^−/−^* macrophages and in vivo upon endotoxin challenge in *Rev-erbα^−/−^* mice [51]. Furthermore, these studies showed that REV-ERBα is a direct transcriptional repressor of several pro-inflammatory genes, including *Ccl2* and NOD-like receptor family pyrin domain containing 3 (*Nlrp3*), which contain RORE binding sites in their promoter regions [56,71]. Moreover, *Rev-erbα^−/−^* mice have been found to display exaggerated LPS-induced pulmonary inflammation [70], increased severity of dextran sulphate sodium (DSS)-induced colitis [56], as well as a neuroinflammatory phenotype with basal activation of microglia in the hippocampus [72]. Likewise, in mice with deficient *Rorα* expression (RORα^sg/sg^, staggerer mutants), several immune defects were described besides typical cerebellar neurodegeneration. For example, splenocytes isolated from these mice were more sensitive to LPS challenge, showing increased expression of pro-inflammatory cytokines compared to wild-type controls [75]. Moreover, similarly to *Rev-erbα* deficient mice, also RORα^sg/sg^ mice showed increased susceptibility to LPS-induced lung inflammation, higher neutrophil numbers, and increased levels of pro-inflammatory cytokines (IL-1β, IL-6, and macrophage inflammatory protein 2) in the bronchoalveolar lavage compared to wild-type mice [74]. An anti-inflammatory action of RORα can occur through RORE-mediated up-regulation of inhibitor of NF-κB (IκBα) and reduced p65 nuclear translocation [82].

REV-ERBα can transcriptionally regulate and repress another circadian repressor NFIL3 [83], which is also implicated in numerous immune processes. Studies in NFIL3-deficient (*Nfil3^−/−^*) mice have shown a critical role of NFIL3 in the development of several types of immune cells, including CD8^+^ conventional dendritic cells [76], NK cells, as well as all other innate lymphoid cell lineages [77,78]. Later, NFIL3 was identified as an important regulator of macrophage responses via transcriptional repression of *Il-12b* [79]. Interestingly, the inflammatory response of macrophages has been shown to depend on the phase of circadian oscillations of NFIL3 and DBP, which competitively bind at the *Il-12b* enhancer [59]. Therefore, desynchronization of the molecular clock in the macrophage population can contribute to the heterogeneity of the inflammatory response [59].

Together, accumulating evidence indicates a complexity of circadian-immune crosstalk, highlighting diverse immunomodulatory effects of individual clock components, which are determined by transcription-dependent mechanisms, direct protein–protein interactions, or the phase of circadian oscillations.

### 4.2. Light-Phase Shifts

Shift work and jet lag represent frequent circadian challenges associated with a modern lifestyle that lead to desynchronization of the SCN and downstream oscillators with the external environment [84]. Shift work refers to work outside the regular daytime hours and involves non-standard work schedules, such as night shifts, early morning shifts, or rotating shifts, which are also associated with alterations in the sleep/wake cycle [85]. Reduced amplitude or disturbance of the key circadian rhythms, such as melatonin, cortisol, and body temperature, has been observed among shift workers [86]. Misalignment between endogenous circadian rhythms and the LD cycle in shift workers can also predispose to an increased risk of negative health outcomes, such as cancer, and metabolic and cardiovascular diseases [87,88]. Moreover, shift workers are at a higher risk of common respiratory infections, including cold, flu, or COVID-19 [89,90,91].

Epidemiological studies revealed increased numbers of total leukocytes, neutrophils, monocytes, and lymphocytes [92,93,94], and reduced activity of NK cells in the circulation of shift workers compared to daytime workers [95]. Moreover, shift workers had significantly elevated markers of systemic inflammation, including C-reactive protein and the cytokines TNFα, IL-6, IL-1β, and IL-10 than daytime workers [94]. One limitation of most observational studies is that they measure these parameters only during the daytime and do not consider their 24 h variability, emphasizing the importance of an interventional approach. In healthy volunteers under laboratory conditions, simulated night shift work protocol with a 10 h delayed sleep period resulted in the reduced amplitude of rhythmic transcripts in peripheral blood mononuclear cells [96] and caused a misalignment of the rhythmic secretion of cytokines IL-6, IL-1β, and TNFα following ex vivo immune stimulation [97].

In animal models, jet lag is induced by single (acute models) or repeated (chronic models) phase delays or advances in the LD cycle [98], while shift work experimental schedules use exposure to contrasting signals, such as light during the dark phase and forced activity or food consumption during the resting period [99]. Chronic jet lag (CJL) protocols have been shown to induce circadian desynchronization in the locomotor activity pattern, which is documented by the appearance of two components of activity rhythms, one with a free-running period and the other with the mean period of a specific shift-lag schedule [100]. Furthermore, CJL modified acrophases of the main clock gene rhythms in the central oscillator, as well as in the peripheral tissues [101].

Experimental studies in mice and rats exploring how circadian disruption induced by light-phase shifts impacts innate immunity and inflammatory responses are summarized in Table 2.

In mice, 24 h following acute jet lag (12 h phase advance), an arrhythmic pattern of circulating blood progenitors [43] and abolished daily variability of leukocyte recruitment to the skeletal muscle were found under both steady state and inflammatory conditions [53]. Similar results were obtained using a CJL protocol. Specifically, jet lag eliminated circadian rhythms in mouse and human blood leukocytes in humanized mice [102] and abolished the rhythm in neutrophil infiltration into the liver, which correlated with increased hepatic accumulation of lipids [103]. The consequences of chronic jet lag on inflammatory responses have been demonstrated in mice using a protocol with 6 h phase advances of the LD cycle every 7 days for 4 weeks followed by 1 week of re-synchronization [104,105,106]. The CJL mice challenged in vivo with a lethal dose of LPS exhibited a higher mortality rate, persistent hypothermia, and amplified serum response of pro-inflammatory cytokines [106]. Corresponding data showing an exaggerated IL-6 response to LPS were also found after ex vivo stimulation of the whole blood or in vitro stimulation of isolated peritoneal macrophages harvested from CJL mice [104,105,106]. Interestingly, LPS-induced IL-6 responses showed a rhythmic pattern in CJL mice, indicating adaptation to a new LD cycle during 1 week of re-synchronization [104]. On the other hand, increased frequency of shifts over one week eliminated time-of-day-dependent mortality rate upon the lethal dose of LPS and enhanced hypothermic and serum TNFα responses in mice [107].

Circadian disruption in shift workers is associated with an increased incidence of metabolic diseases. Macrophages represent key mediators of obesity-induced inflammation in mice and humans [113,114]. In mice on a high-fat diet (HFD), chronic exposure to shifts of the LD cycle amplified adipose tissue macrophage infiltration and pro-inflammatory M1 polarization, together with enhanced expression of pro-inflammatory cytokines [108]. Similarly, in hyperlipidemic APOE*3-Leiden.CETP mice on an HFD, circadian disruption induced by weekly LD reversals over several weeks accelerated the development of atherosclerosis, increased the macrophage content in atherosclerotic lesions, and promoted a pro-inflammatory state in the vessel wall [109].

Circadian clocks have been studied as an important player in many aspects of cancer-immune cell interactions [115]. Experimental research has shown that circadian disruption induced by different jet lag models can accelerate tumor growth and the incidence of metastasis as compared to a normal lighting regime [110,111,112,116]. Innate lymphoid NK cells are an integral part of anti-tumor immunity and provide effective immune surveillance by destroying tumor cells [117]. This ability is ensured by a stable count of NK cells and their production of various cytolytic factors and cytokines, mainly perforin, granzyme B, and IFNγ [118,119]. In mice, chronic shifts in the LD cycle reduced the numbers of NK cells in the spleen and lungs [111] and attenuated their cytolytic activity through suppressed expression of CD107a, a sensitive indicator of NK cell cytotoxicity and degranulation [120]. Another study in rats showed that repeated phase advances of the LD cycle suppressed rhythmic cytotoxicity of splenic NK cells, and modified circadian expression of granzyme B, perforin, and IFNγ in NK cells [112]. In addition to NK cells, tumor progression is controlled by the tumor microenvironment, which contains a variety of immune cells with a tumor-promoting or tumor-suppressing phenotype [121]. In a melanoma mouse model, circadian disruption induced by CJL abolished daily variability and decreased the M1 (pro-inflammatory)/M2 (anti-inflammatory) macrophage ratio in the tumor, promoting immunosuppression of the tumor microenvironment [110]. These effects accelerated tumor growth, and were also associated with increased mortality [110]. Other studies found reduced survival in aged mice exposed to chronic phase-advances for 8 weeks [122] or even as a result of long-term exposure (for 85 weeks) to phase-advances in 4-day intervals [123]. Additionally, epigenetic changes are known to participate in carcinogenesis, and they can also have the potential to mediate deregulation of immune mechanisms induced by circadian disruption. For example, rats, experienced chronic circadian disruption, exhibited aberrant changes in the expression of several cancer-related microRNAs in mammary tissues and, these changes were associated with increased protein levels of pro-inflammatory transcription factors, phosphorylated NF-κB and STAT3 [124].

### 4.3. Dim ALAN

The advancement of lighting technologies, including the implementation of light-emitting diode (LED) technology, goes in parallel with increasing levels of light pollution [125]. Moreover, evening use of devices with light-emitting screens as well as the use of night lamps, especially for small children while sleeping, considerably contribute to unintentional exposure to ALAN [126].

Evidence provided by experimental studies has demonstrated that dim ALAN (≤5 lx) can compromise circadian coordination in laboratory rodents. The rhythmic profile of locomotor activity was preserved in rats exposed to dim ALAN for 2 weeks, but mean night-time levels were reduced, and daytime activity was increased compared to controls [127]. Another study in rats reported that dim ALAN diminished the power of 24 h activity rhythm and induced a second approximately 25 h free-running rhythm, indicating internal desynchronization of locomotor activity [128]. In the SCN, dim ALAN exposure clearly suppressed the daily rhythms of clock genes in both rats [129,130] and mice [131,132]. In peripheral tissues, clock gene rhythms appeared to be less affected by ALAN than in the master oscillator, though they showed lowered amplitude or shifts in acrophase [127,129,131]. The daily plasma melatonin rhythm was eliminated in rats after 2 weeks of dim ALAN (2 lx) exposure due to suppressed nocturnal melatonin levels [129], which were also reported in other studies, not only in rats [133,134] but also in diurnal birds [135,136] and humans [137]. Moreover, circadian disruption induced by dim ALAN has been observed in other hormonal rhythms, e.g., suppressed and phase-advanced corticosterone rhythm, and abolished daily rhythmicity in plasma testosterone and vasopressin levels in rats [129].

Several experimental studies have demonstrated that ALAN can affect innate immune mechanisms, including inflammatory response (Table 3). However, in most of these studies, the immune status was evaluated only at one time point, neglecting consequences on circadian rhythms in the immune system. Indeed, a recent study showed that rats exposed to dim ALAN (2 lx) for 5 weeks exhibited impaired daily variation of the main leukocyte subsets in the blood, especially monocytes and T cells [138]. Moreover, ALAN reduced blood monocyte counts and altered gene expression of macrophage marker *Cd68* and chemokine *Ccl2* in the kidney, indicating that weakened circadian control of circulating leukocyte numbers was associated with disturbed renal immune homeostasis [138].

Immune disbalance caused by ALAN is considered one of the key mechanisms that can promote a pro-inflammatory state or accelerate various pathologies. For example, mice exposed to either ALAN (5 lx) or an HFD for 4 weeks showed up-regulated expression of inflammatory markers *Tnfα* and macrophage-1 antigen (*Mac-1*) in white adipose tissue, while ALAN further potentiated HFD-induced inflammation [139]. In cancer research, dim ALAN has been shown to favor tumor growth, especially in models of mammary cancer [143,144]. C3H mice exposed to ALAN (5 lx) for 3 weeks and then injected with FM3A mammary carcinoma cells displayed earlier tumor onset and increased terminal tumor volume compared to tumor-bearing mice housed in the LD regime [143]. In another study in nude rats, chronic ALAN even with a very low light intensity of 0.2 lx accelerated mammary tumor growth [144].

Another process that can drive the impact of ALAN on the progression of diseases is the ability of ALAN to promote neuroinflammation. Exposure to ALAN for 4 weeks increased hippocampal *Tnfα* and *Il-6* expression simultaneously with depression-like behavior in female Siberian hamsters (*Phodopus sungorus*) [145], and up-regulated *Il-6* mRNA levels in the medulla of mice that concomitantly exhibited cold hyperalgesia and mechanical allodynia [140]. Moreover, mice that underwent global cerebral ischemia and were subsequently exposed to ALAN showed decreased survival associated with increased neuronal damage that was preceded by amplified neuroinflammation, compared to animals in the control regime [142].

Till now, the effects of ALAN on inflammatory response were examined only in a limited number of studies. In mice challenged with LPS following 4 weeks of dim ALAN (5 lx), exaggerated changes in body temperature, prolonged sickness responses, and elevated pro-inflammatory cytokine expression (*Tnfα* and *Il-6*) in microglia were found compared to controls [141]. Additionally, diminished bactericidal capacity of blood upon LPS challenge and reduced delayed-type hypersensitivity response was observed in Siberian hamsters exposed to dim ALAN compared to animals in the standard LD regime [146]. Interestingly, the opposite effects of ALAN were obtained in a diurnal rodent model, Nile grass rats (*Arvicanthis niloticus*), which exhibited enhanced delayed-type hypersensitivity response and elevated basal bactericidal capacity when exposed to ALAN for 3 weeks [148]. Thus, the currently available data demonstrate that ALAN affects the responsiveness of the immune system to challenges, but clearly more studies are needed to reveal potentially differential responses between diurnal and nocturnal mammals, and to evaluate whether immune responses are impacted by ALAN in a time-of-day-dependent manner. Moreover, surprisingly limited data are available on the effects of ALAN on innate immunity and inflammation in humans.

### 4.4. Constant Light

Exposure to LL and low-intensity ALAN are often considered interchangeable conditions. However, circadian disruption caused by LL differs from that induced by low-intensity ALAN in several ways [149]. In general, LL leads to the complete loss of locomotor activity rhythms [150], and this behavioral arrhythmicity develops as soon as one month after changed lighting conditions in rats [151,152]. In the master clock, LL causes desynchronization of SCN neurons [150] and reduces the amplitude of SCN neuronal activity rhythm [153], which is further attenuated by long-term LL exposure [154]. Suppressed nocturnal melatonin levels have been found under both LL and dim ALAN regimes [155,156] but corticosterone is arrhythmic in LL [133,157], and preserves its rhythmicity with decreased amplitude in the dim ALAN regime [129].

The effects of LL exposure on immune functions have been reported by several studies, which are summarized in Table 4. Circadian disruption induced by LL was shown to facilitate a pro-inflammatory state even under unchallenged conditions. Specifically, in rats, 4-week LL exposure up-regulated the expression of the pro-inflammatory markers *Stat3*, *Il-17ra*, and *Il-1α* in the colonic mucosa [151]. In another study, rats exposed to LL for 5 weeks displayed amplified plasma TNFα response and sickness symptoms, such as febrile reaction and food intake reduction, following LPS administration [152]. Interestingly, in rats, 24 h leukocyte rhythms in the circulation persisted 8 weeks after LL exposure, despite suppressed circadian rhythms in body temperature and locomotor activity [158]. Nevertheless, in the same study, prolonged LL exposure (for 11 and 16 weeks) did eliminate the circadian rhythm in blood leukocytes that was not restored even 16 weeks after re-synchronization in the LD regime [158]. In mice exposed to LL for 8 weeks, increased numbers of blood neutrophils and reduced numbers of lymphocytes were found together with an enhanced response of pro-inflammatory cytokines to LPS challenge [154]. Interestingly, these effects were transient, as no further changes were observed in mice after 24 weeks of the LL regime [154]. However, the study did not monitor the whole 24 h profile in white blood cells.

The disruption of circadian rhythms due to LL has also been demonstrated to promote tumorigenesis and adversely affect chronic inflammatory processes [152,159,160]. Rats implanted with C6 tumor cells and exposed to LL for 5 weeks exhibited faster tumor growth and increased tumor infiltration with macrophages compared to controls in the LD regime [152]. The mechanisms behind these effects are not clear, but may involve reduced NK cell counts, also found in rats exposed to LL [158]. In addition, recent evidence suggests that myeloid-derived suppressor cells are associated with a poor prognosis in cancer [161]. In the mouse model of chronic inflammation, 4-week LL potentiated the accumulation of myeloid-derived suppressor cells (granulocytic CD11b^+^Ly6G^high^ and monocytic CD11b^+^CD49d^+^ cell subsets) in the spleen and elevated IL-6 levels in the circulation [160].

## 5. Conclusions

**Figure 1:**
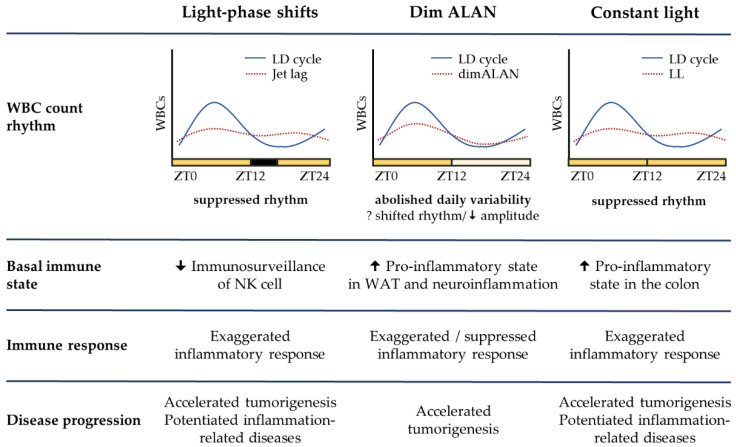
Comparison of three disruptive light/dark regimes and their effects on the immune measures based on the current knowledge in laboratory animals. ALAN—artificial light at night; LD—standard light/dark cycle of 12/12 h; LL—constant light; WAT—white adipose tissue; WBCs—white blood cells; ZT—Zeitgeber time.

Chronodisruptive risk factors, such as mistimed light information due to shift work or ALAN exposure, are not associated with acute pain, increasing the chance that their negative health consequences will be overlooked. Innate immune cells represent the first line of defense against pathogenic stimuli. The effector functions of innate immune cells are profoundly controlled by cell-intrinsic molecular clocks, which can coordinate immune cell trafficking and the production of immune-regulatory molecules in a time-of-day-dependent manner. Accumulating evidence from experimental studies in mice and rats demonstrates that disruptive LD regimes can compromise these surveillance mechanisms and shift the immune balance to a pro-inflammatory state. Indeed, the obvious effects, observed under light-phase shifts, dim ALAN, and LL, are represented by exaggerated acute inflammatory response upon LPS challenge, promoted tumorigenesis, and amplified symptoms associated with chronic inflammation (Figure 1).

These effects can result from disrupted immune rhythms and disrupted circadian gating of immune responses, though there is still a lack of experimental data, particularly under dim ALAN exposure. Moreover, existing studies rarely specify other details regarding the quality of light than light intensity, and this can be critical for the evaluation of adverse health consequences.

Collectively, a better understanding of the mechanisms by which circadian disruption influences the immune status can be of importance in the search for strategies to prevent or limit the negative consequences of chronodisruption on health.

## Figures and Tables

**Table 1:** Animal models with genetic disruption in specific clock components and their effects on processes related to innate immune functions and inflammation.

Genotype	Immune Challenge	Effects	Refs.
*Bmal1^−/−^* mice (global KO)		Lost daily rhythms in the circulating numbers of white blood cells and their progenitors	[43,54]
*Bmal1^−/−^* mice (global KO)	KLA (in vitro)	Disturbed transcriptome response to TLR4 activation in BMDMs (enhanced and prolonged response of *Il-1β*, *iNos* and *Hif1α*)	[55]
*Bmal1^−/−^* mice (global KO)		↑ severity of DSS-induced colitis	[56]
*Arntl^LoxP^/^LoxP^Lyz2^Cre^* mice (myeloid-specific *Bmal1 KO)*		Lost daily variability in Ly6C^high^ monocyte counts in the blood, spleen, and bone marrow	[52]
*Arntl^LoxP^/^LoxP^Lyz2^Cre^* mice (myeloid-specific *Bmal1 KO)*	TG-induced peritoneal inflammation	↑ peritoneal recruitment of Ly6C^high^ monocytes and amplified CCL2, CCL8, IL-1β, and IL-6 response	[52]
*Arntl^LoxP^/^LoxP^Lyz2^Cre^* mice (myeloid-specific *Bmal1 KO)*	*Listeria monocytogenes* infection	↓ survival and ↑ serum levels of IL-1β, IL-6, IFNγ, and CCL2	[52]
*Bmal1^−/−^Lys-MCre* mice (myeloid-specific *Bmal1* KO)	LPS 25 mg/kg (i.p.)	Lost protection to LPS-induced lethality at ZT0 compared to ZT12	[57]
*Bmal1^−/−^Lys-MCre* mice (myeloid-specific *Bmal1* KO)	LPS 100 ng/mL (in vitro)	↑ LPS-induced production of IL-6, TNFα, CXCL1 and CCL2 and ↓ levels of IL-10 in BMDMs ↑ pro-inflammatory microRNA miR-155 induction upon LPS in BMDMs	[57]
*Bmal1^LoxP^/^LoxP^Lyz2^Cre^* mice (myeloid-specific *Bmal1* KO)	LPS 100 ng/mL (in vitro) or LPS 5 mg/kg (i.p.)	↓ NRF2 response in LPS stimulated BMDMs ↑ basal and LPS stimulated ROS levels, ↑ LPS stimulated IL-1β and HIF1α levels in BMDMs ↑ serum IL-1β response to in vivo LPS stimulation	[58]
*Bmal1^FloxP/FloxP^;LysM^Cre^ mice* (myeloid-specific *Bmal1* KO)	LPS 10 or 100 ng/mL (in vitro)	Lost daily variability in IL-12p40-producing cells in LPS-stimulated peritoneal macrophages	[59]
*LysM-Bmal1^−/−^* mice (myeloid-specific *Bmal1* KO)	*Streptococcus pneumoniae* or *Staphylococcus aureus* infection	Protection against pneumococcal infection ↑ phagocytic activity in peritoneal and alveolar macrophages	[60]
*LysM-Bmal1^−/−^* mice (myeloid-specific *Bmal1* KO)	LPS 1 mg/kg (i.p.)	Lost daily variability in IL-6 response to LPS in peritoneal macrophages	[51]
*Bmall^LoxP^/^LoxP^Lyz2^Cre^* mice *(ApoE^−/−^ background)* (myeloid-specific *Bmal1* KO)		↑ size of atherosclerotic lesions ↑ recruitment of Ly6C^high^ monocytes and accumulation of pro-inflammatory M1 macrophages in atherosclerotic lesions	[61]
*Bmal1*^ΔN^ mice (neutrophil-specific deletion of *Bmal1*)		Lost daily variability in neutrophil proteome, granule content and NET formation	[62]
*Clock^−/−^* mice (global KO)	TNFα 2 ng/mL or CBLB502 100 ng/mL (in vitro)	↓ NF-κB activation upon TNFα treatment in MEFs and upon bacterial flagellin (CBLB502) treatment in hepatocytes	[49]
*Clock* mutant mice	LPS 1 µg/mL or *S. Typhimurium* (in vitro)	↓ expression of pro-inflammatory genes *Il-6*, *Il-1β*, *Tnfα*, *Cxcl1*, *Ifnβ*, and *Ccl2* and ↓ TNFα and IL-6 response in BMDMs	[63]
*Clock* mutant mice	*Salmonella* infection (in vivo)	Impaired rhythmicity in bacterial colonization in the gut and reduced pro-inflammatory gene expression	[63]
*Clock* mutant mice	LPS 1 µg/mL (in vitro)	↓ LPS-induced expression of *Il-6*, *Il-1β* and *Cxcl1* in MEFs ↑ RELB and p100/52 protein levels in MEFs independent of LPS	[64]
*Per1^tm1Drw^* mutant mice		Modified circadian rhythms of perforin, granzyme B and IFNγ in the splenic NK cells	[65]
*mPer2^Brdml^* mutant mice		Lost daily IFNγ rhythms (splenic mRNA and protein expression, and serum levels) in the spleen	[66]
*mPer2^Brdml^* mutant mice	LPS 25 mg/kg (i.p.)	↑ survival upon lethal dose of LPS and suppressed daily rhythm in susceptibility to endotoxic shock ↓ serum IFNγ and IL-1β levels and ↓ IFNγ production by splenic NK cells in response to LPS	[67]
*mPer2^Brdml^* mutant mice	TLR9 ligand (in vitro)	↓ TNFα and IL-12 production in challenged peritoneal macrophages and ↓ *Tlr9* expression	[68]
*Cry1^−/−^Cry2^−/−^* mice and fibroblasts (double KO)		Constitutive activation NF-κB via PKA signaling in fibroblasts ↑ constitutive expression of pro-inflammatory molecules in the hypothalamus and fibroblasts (*Il-6*, *Tnfα* and *iNos*), and in the BMDMs (*Il-6*, *Cxcl1* and *iNos*) ↑ inflammatory response of BMDMs to LPS (TNFα and IL-6)	[69]
*Rev-erbα^−/−^* mice (global KO)	LPS 1 mg/kg (i.p.) LPS 1 µg/mL (in vitro)	Lost circadian response of IL-6 to LPS challenge in vivo and in vitro using isolated PECs	[51]
*Rev-erbα^−/−^* mice (global KO)	aerosolized LPS 2 mg/mL	↑ neutrophil numbers and CXCL1, CXCL2 and CXCL5 levels in BAL fluid	[70]
*Rev-erbα^−/−^* mice (global KO)	LPS 100 ng/mL (*ex vivo*)	↑ cytokine and chemokine response to LPS (*Il-6*, *Ccl2* and *Ccl5* expression) in alveolar macrophages	[70]
*Rev-erbα^−/−^* mice (global KO)	LPS 1 µg/mL (in vitro)	↑ basal and LPS-stimulated *Ccl2* gene expression in peritoneal macrophages	[71]
*Rev-erbα^−/−^* mice (global KO)		↑ basal NF-κB signaling and pro-inflammatory microglial activation in the hippocampus ↑ LPS-induced neuroinflammation	[72]
*Rev-erbα^−/−^* mice (global KO)		↑ complement transcripts (C4b and C3) in the hippocampus	[73]
*Rev-erbα^−/−^* mice (global KO)	DSS-induced colitis	↑ severity of DSS-induced colitis ↑ colonic levels of NLRP3, IL-1β, and IL-18 in DSS-induced colitis suppressed daily rhythm of *Nlrp3* in the colon	[56]
*Rev-erbα^−/−^* mice (global KO)	LPS 100 ng/mL (in vitro)	↑ LPS-induced protein levels of NLRP3 and IL-1β in peritoneal macrophages	[56]
staggerer (RORα^sg/sg^) mice	intra-tracheal LPS 2 µg/50 µL	↑ susceptibility to LPS-induced airway inflammation ↑ neutrophil counts and cytokine levels IL-1β, IL-6, and MIP-2 in BAL fluid	[74]
staggerer (RORα^sg/sg^) mice	LPS 5 µg/mL (in vitro)	↑ *Il-1β, Il-1α*, and *Tnfα* expression in LPS-stimulated splenocytes	[75]
*Nfil3^−/−^* mice (global KO)		Lack of CD8α^+^ cDC population in the lymphoid organs	[76]
*Nfil3^−/−^* mice (global KO)		Lack of NK cells and impaired NK-cell mediated cytotoxicity	[77]
*Nfil3^−/−^* mice (global KO)	*Clostridium difficile* infection	↓ numbers of innate lymphoid cells in the intestinal mucosa ↓ immune defence against acute intestinal bacterial infection with *Clostridium difficile*	[78]
*Nfil3^−/−^* mice (global KO)	LPS 10 ng/mL (in vitro)	↑ LPS-induced *Il-12b* expression and IL-12p40 release from BMDMs Spontaneous expression of *Il-12b* in colonic CD11b^+^ LPMCs	[79]
*Nfil3^−/−^* mice (global KO)	LPS 10 ng/mL (in vitro)	↑ proportion of IL-12p40 producing macrophages in response to LPS and ↑ expression of *Ccr2* in unstimulated BMDMs	[59]

APOE—apolipoprotein E; BAL-bronchoalveolar lavage; BMDMs—bone marrow-derived macrophages; CCL2/5/8—CC motif chemokine ligand 2/5/8; cDC—conventional dendritic cells; CXCL1/2/5—CXC motif chemokine ligand 1/2/5; DSS—dextran sulphate sodium; HIF1α—hypoxia-inducible factor 1α; IFNγ—interferon gamma; IL—interleukin; iNOS—inducible nitric oxide synthase; KLA—Kdo2-lipid A (TLR4 ligand); KO—knock-out; LPMCs—lamina propria mononuclear cells; LPS—lipopolysaccharide; MEFs—mouse embryonic fibroblasts; MIP-2—macrophage inflammatory protein 2; NET—neutrophil extracellular trap; NF-κB—nuclear factor kappa B; NLRP3—NOD-like receptor family pyrin domain containing 3; NRF2—nuclear factor-like 2; PECs—peritoneal exudate cells; PKA—protein kinase A; RELB—subunit of nuclear factor kappa B; ROS—reactive oxygen species; TG—thioglycolate; TLR—toll-like receptor; TNFα—tumor necrosis factor alpha; ZT—zeitgeber time.

**Table 2:** The effects of different light-phase shift paradigms on the immune parameters and functions under the steady state and challenged conditions in rodents.

Species	Shift Paradigm	Immune Challenge	Effects	Ref.
Humanized NSG mice	8 h PA/2 days for 10 days		Eliminated circadian rhythm of mouse and human blood leukocytes	[102]
Lyzs-Cre mice	LD reverse/5 days for 3 weeks		Eliminated circadian rhythm of neutrophil hepatic infiltration and ↑ triacylglycerol levels in the liver	[103]
C57BL/6J mice	6 h PA/7 days for 4 weeks and 1 week of re-synchronization	LPS 50 μg/mL (ex vivo) or 1 µg/mL (in vitro)	↑ LPS-induced IL-6 response of the whole blood and incubated PECs, preserving a daily rhythm in this immune response	[104] [105]
C57BL/6J *Per2^Luc^* mice	6 h PA/7 days for 4 weeks and 1 week of re-synchronization	LPS 10 µg/mL (in vitro)	↑ LPS-induced IL-6 response of incubated PECs	[106]
C57BL/6J *Per2^Luc^* mice	6 h PA/7 days for 4 weeks and 1 week of re-synchronization	LPS 12.5 mg/kg (i.p.)	Persistent hypothermia, ↑ mortality rate, ↑ response of pro-inflammatory cytokines (IL-1β, GM-CSF, IL-12, IL-13) to LPS	[106]
C57BL/6J mice	6 h PA/2 days for 3 weeks	LPS 20 mg/kg (i.p.)	80% mortality rate independent of time of LPS administration, ↑ hypothermic and serum TNFα response	[107]
C57BL/6J *Per2^Luc^* mice on HFD	LD reverse/5 days for 10 weeks		↑ adipose tissue macrophage infiltration and pro-inflammatory M1 polarization associated with amplified expression of *Il-1β*, *Il-6* and *Tnfα* ↑ pro-inflammatory activation of BMDMs with ↑ LPS-induced expression of *Il-1β*, *Il-6*, and *Tnfα*	[108]
APOE*3-Leiden.CETP mice on HFD	LD reverse/7 days for 10 and 15 weeks		↑ atherosclerosis development in the aortic root ↑ lesion macrophage content and ↑ vascular expression of markers for inflammation (*Nfκb1*, *Tnfα*, *iNos*), oxidative stress (*Sod1*, *Gpx1*, *Hif1α*, *Nox2*), and leukocyte recruitment (*Icam1*, *Ccr2*, CCL2) at ZT0 Phase-shifted rhythms of circulating leukocytes	[109]
C57BL/6J mice	8 h PA/2–3 days for 8 weeks		↑ severity of DSS-induced colitis	[56]
C57BL/6J mice	6 h PA/2 days for 3 weeks	B16F0 nonmetastatic melanoma cells (s.c.)	↑ mortality rate, ↑ tumor growth rate, lost daily variability and M1/M2 macrophage ratio in melanoma tumors	[110]
C57BL/6J mice	LD reverse/4 days for 12 weeks		↓ NK cell numbers in the spleen and lungs ↓ expression of CD107a and IFNγ in non-stimulated and activated splenic NK cells ↑ lung metastasis of B16 melanoma	[111]
Fischer rats	6 h PA/2 days for 3 weeks and 1 week in DD		↓ rhythm and cytotoxicity of splenic NK cells ↓ or shifted circadian rhythms of perforin, granzyme B and IFNγ in NK cells	[112]
Fischer rats	6 h PA/2 days for 3 weeks and 1 week in DD	MADB106 tumor cells (i.v.)	↑ lung tumor frequency (after 6–8 weeks in LD) and ↑ cytolytic activity of NK cells (24 h post stimulation)	[112]

BMDMs—bone marrow-derived macrophages; CCL2—CC motif chemokine ligand 2; CCR2—CC chemokine receptor 2; DD—constant darkness; DSS—dextran sulphate sodium; GM-CSF—granulocyte-macrophage colony-stimulating factor; GPX1—glutathione peroxidase 1; HFD—high fat diet; HIF1α—hypoxia-inducible factor 1α; ICAM1—intercellular adhesion molecule 1; IFNγ—interferon gamma; IL—interleukin; iNOS—inducible nitric oxide synthase; LD—light/dark; LPS—lipopolysaccharide; NF-κB1—nuclear factor kappa B; NOX2—NADPH oxidase 2; PA—phase advance; PECs—peritoneal exudate cells; SOD1—superoxide dismutase type 1; TNFα—tumor necrosis factor alpha; ZT0—beginning of the light phase.

**Table 3:** Summary of the effects of dim artificial light at night (ALAN) on the immune parameters under steady state and challenged conditions in rodents.

Species	ALAN Intensity and Duration	Immune Challenge	Effects	Ref.
Wistar rats	L: 150 lx; dimL: 2 lx for 2 and 5 weeks		Impaired daily variation in the numbers of circulating monocytes and T cells ↓ numbers of blood monocytes ↑ expression of macrophage marker *Cd68* and ↓ *Ccl2* expression in the kidney	[138]
Swiss Webster mice	L: 150 lx; dimL: 5 lx for 4 weeks		↑ expression of *Mac-1* and *Tnfα* in WAT Exacerbated peripheral inflammation associated with HFD	[139]
CFW mice	L: 125 lx; dimL: 5 lx for 4 weeks		↑ *Il-6* expression in the medulla associated with cold hyperalgesia and mechanical allodynia	[140]
Swiss Webster mice	L: 150 lx; dimL: 5 lx for 4 weeks	LPS 0.5 mg/kg (i.p.)	Exaggerated changes in body temperature and prolonged sickness responses to LPS ↑ LPS-induced expression of *Tnfα* and *Il-6* in microglia	[141]
Swiss Webster mice	L: 150 lx; dimL: 5 lx for 1 week	Model of global cerebral ischemia	↑ mortality rate 7 days following injury ↑ neuroinflammation 24 h following injury (amplified *Tnfα* mRNA levels in the hippocampus)	[142]
C3H mice	L: 150 lx; dimL: 5 lx for 3 weeks	FM3A mammary carcinoma cells	↓ latency to tumor onset and ↑ tumor volume	[143]
Nude rats	L: 345 lx; dimL: 0.2 lx for 6 weeks	MCF-7 human breast cancer xenografts	↑ tumor growth rate	[144]
Siberian hamsters	L: 150 lx; dimL: 5 lx for 4 weeks		↑ *Tnfα* and ↓ *Bdnf* expression in the hippocampus ↓ hippocampal dendritic spine density	[145]
Siberian hamsters	L: 150 lx; dimL: 5 lx for 4 weeks	LPS 0.4 mg/kg (i.p.) or DNFB treatment	↓ plasma bactericidal capacity following LPS ↓ delayed-type hypersensitivity response to DNFB	[146]
Nile grass rats	L: 150 lx; dimL: 5 lx for 3 weeks		↑ basal plasma bactericidal capacity ↑ delayed-type hypersensitivity response to DNFB	[147]

BDNF—brain-derived neurotrophic factor; CCL2—CC motif chemokine ligand 2; dimL—dim light phase; DNFB—2,4-dinitro-1-fluorobenzene; HFD—high fat diet; IL—interleukin; L—light phase; LPS—lipopolysaccharide; MAC-1—macrophage-1 antigen; TNFα—tumor necrosis factor-alpha; WAT—white adipose tissue.

**Table 4:** Summary of the effects of constant light (LL) on the immune parameters under steady state and challenged conditions in rodents.

Species	LL Intensity and Duration	Immune Challenge	Effects	Ref.
Sprague-Dawley rats	300 lx for 17 weeks and 16 weeks of re-synchronization		Lost 24 h rhythm in blood leukocytes after 11/16 weeks in LL, (the rhythm was not restored after 16 weeks of re-synchronization in LD regime) ↓ NK cell counts in the blood	[158]
Wistar rats	150 lx for 4 weeks		Activated pro-inflammatory state (↑ expression of *Stat3*, *Il-1α* and *Il-17ra*) in the colonic mucosa	[151]
Wistar rats	200–250 lx for 5 weeks	LPS 2 µg/kg (i.v.)	↑ plasma TNFα response and sickness symptoms upon LPS	[152]
Wistar rats	200–250 lx for 5 weeks	C6 tumor cells (s.c.)	↑ tumor growth ↑ tumor infiltration of monocytes/macrophages	[152]
Sprague-Dawley rats	200 lx for 7 days	Endotoxemia model (daily i.p. LPS injection for 7 days)	↑ hypothalamic expression of *Il-1β* and *Tnfα*	[159]
C56BL/6J mice	105 lx for 24 weeks	LPS 50 µg/kg (i.v.)	Transient ↑ of neutrophil and ↓ of lymphocyte numbers in the blood was associated with enhanced inflammatory response to LPS (↑ IL-1β, TNFα, IL-6, and ↓ IL-10 plasma levels) after 8 weeks of LL No immune changes after 24 weeks of LL	[154]
CD-1 mice	750 lx for 4 weeks	Complete Freund’s adjuvant (100 µL)	↑ proportion of myeloid-derived suppressor cells in the spleen under LL was potentiated by chronic inflammation ↑ plasma TGF-β1 levels and ↑ chronic inflammation induced elevation of IL-6 levels	[160]

IL—interleukin; LD—light/dark; LPS—lipopolysaccharide; STAT3—signal transducer and activator of transcription 3; TGF-β1—transforming growth factor beta 1; TNFα—tumor necrosis factor-alpha.
